# Bio-Based Healable 2K PU Textile Coating for Durable Applications

**DOI:** 10.3390/polym14194014

**Published:** 2022-09-25

**Authors:** David De Smet, Myriam Vanneste

**Affiliations:** Centexbel, Technologiepark 70, 9052 Zwijnaarde, Belgium

**Keywords:** polyurethane (PU), healing, coating, textile, bio-based

## Abstract

A biobased healable 2K polyurethane (PU) coating incorporating a Schiff base was synthesized and applied as a thin coating on textiles. The Schiff base, made out of cystine and vanillin, contained reversible imine and disulfide bonds and was used as a chain extender in PU synthesis. The FT-IR analysis indicated the successful incorporation of the Schiff base in the PU backbone. Compared with control PU coatings, the healable bio-based PU coating with the Schiff base showed very good healing properties using heat as external stimuli: a healing recovery of 75% was obtained after applying a 2 N scratch and complete recovery of the resistance to hydrostatic pressure. SEM analysis revealed complete closure of the scratch after healing for 30 min at 90 °C. The healing properties are attributed to the synergy of the dual-dynamic metatheses of the imine and disulfide bonds.

## 1. Introduction

Polyurethane (PU) is one of the most used polymers in the world, especially in coatings, adhesives, sealants and elastomers (CASE applications) but also foams. Indeed, PU has properties that can be tailored according to the application, such as high flexibility, good tear strength, good abrasion and chemical resistance. To increase the lifetime of polymers, such as PU, healable and recyclable polymers have been developed based on extrinsic healing or intrinsic healing. Extrinsic healing is obtained by embedding a healing agent in a polymer matrix. Upon a crack in the matrix, the healing agents will be released to close the crack. An example is the use of microcapsules containing a healing agent in textile coatings. This is a single-use nonreversible process and since extrinsic healing lacks repetitive healing capacity, more attention is paid to intrinsic healing. Intrinsic healing refers to reversible noncovalent and/or dynamic covalent cross-linking in polymer networks. After dissociation, noncovalent crosslinked networks can be reorganized and reformed upon the interdiffusion of polymer chains due to the reversible break-reformation potential of noncovalent linkages. However noncovalent healing often results in a decrease in mechanical performance, limiting their application. Dynamic covalent crosslinked networks can be produced by crosslinking pre-polymers and small molecules (e.g., chain extender) via dynamic covalent bonds. These materials need external stimuli (often heat) to be healable [1,2,3,4,5,6].

Noncovalent interactions are weaker compared to covalent bonds and include hydrogen bonding, electrostatic, metal-coordination, host-guest and ion-dipole interactions. A healable PU elastomer was synthesized based on polypropylene carbonate, 1,6-hexamethylendiamaine and 4,4′-diphenyl methane diisocyanate. Non-covalent van der Waals and hydrogen bonding are the driving force for the healing of the elastomer. The healing efficiency varied between 44% and 100% depending on the type of PU and the temperature. The best results were obtained at 50 °C [7]. A blend of linear and branched PU’s having triazole ligand end groups, non-covalently linked through Fe/triazole interaction, were developed. The materials exhibited a healing efficiency of over 90% [8]. A supramolecular healable PU elastomer based on aromatic π–π stacking and hydrogen bonds is described. Healing was obtained by heating at 45 °C. The mechanical properties were characterized, and the healing efficiency was approximately 99% [9]. The development of zwitterionic multi-shape-memory PU with healing properties as potential smart biomaterials was described [10]. Self-healing thermoset PU based on cyclodextrin and adamantane host–guest interaction was reported and exhibited healing at room temperature. Healing efficiency increased as the content of the host-guest moieties increased [11].

In the case of dynamic covalent bonds, one can distinguish between dissociative and associative bonds. During the dissociative process, cross-linkers are dissociated into their individual reactive constituent partners before reforming, while in the case of an associative process a substitution reaction occurs between an existing cross-link and pendent reactive group. Examples of dissociative dynamic covalent bonds include disulfide and diselenide bonds, Diels-Alder adducts and boroxines. Transamination, transesterification or silyl ether exchange are the most occurring associative mechanisms [12,13].

Vitrimers are cross-linked materials showing an associative dynamic bond-exchange mechanism resulting in topology alternation. The first vitrimer reported was a carboxylic acid-cured epoxy network through transesterification under catalytic Zn(OAc)2 [14]. A non-isocyanate polyurethane vitrimer was developed, exhibiting healing through transcarbonation exchange reactions at 130 °C. The healing efficiency was 88% [15]. Transamination of vinylogous urethanes was studied as an exchange reaction for catalyst-free vitrimers [16]. A PU-modified epoxy vitrimer with amino ester moieties was prepared. Scratches on the surface of the samples could be healed within 30 min at 200 °C based on transesterification [17].

Next to associative mechanisms, dissociative-based healing mechanisms are extensively explored for PU networks. A bio-based Diels-Alder adduct made out of CO_2_ and furfuryl was reported, having a healing efficiency of 94% when it was healed at 120 °C for 10 min and then healed at 60 °C for 24 h [18]. A healing PU powder coating system containing a commercial uretdione-based cross-linker (BF1320), OH-functionalized polyester resin, and a Diels–Alder adduct as a healing agent is reported. The system exhibited 100% healing of a crack within 12.5 min at 120 °C [19]. Behera et al. reported a dual-functional (PU) elastomer having disulfide as well as furfuryl functionalities to introduce healing properties. The PU showed high healing efficiency of 97% and high tensile strength (39.5 MPa) [20]. A bio-based Schiff base was synthesized out of vanillin and cystine and used as a cross-linker in PU. The Schiff base consisted of dynamic covalent disulfide and imine bonds resulting in a PU with high healing efficiency (97%). Self-healing was obtained via heat or UV irradiation [21]. A transparent healable PU based on disulfide bonds, using 4-aminophenyl disulfide as a chain extender was synthesized. The healing efficiency was up to 93% after 24 h at 80 °C. In the case of PU without disulfide bonds, shape memory behavior caused crack closure, but no intrinsic self-healing component through dynamic disulfide bond exchange occurred, leading to low healing efficiency (36%) [22]. A healable PU dispersion incorporating disulfide bonds was synthesized. The healing efficiency reached 96% after 4 h at 70 °C and more than 80% after 24 h at 25 °C. However, no comparison was made with PU without disulfide bonds [23]. A PU dispersion using cystamine as a chain extender was developed. The healing efficiency was 40% after 3 h at 130 °C, while the corresponding PU dispersion using ethylene diamine as chain extender, and thus without disulfide bonds, exhibited no healing [24]. By incorporating dynamic diselenide bonds into PU, visible light-induced self-healing elastomers were developed. Depending on the composition of the PU elastomer the self-healing efficiency could reach 72% after 48 h visible light irradiation. For the control sample with no diselenide bonds, almost no healing occurred after even 72 h visible light irradiation [25]. Moreover, aromatic diselenide crosslinkers were used to enhance the reprocessability and healing of PU thermosets. It was demonstrated that aromatic diselenides have lower bond energy than their disulfide counterparts and exchange faster. An aromatic diselenide has been incorporated into PU using a para-substituted amine diphenyl-diselenide. After 30 min PU samples based on diselenide showed 56% healing efficiency of their initial mechanical properties, while corresponding disulfide PU samples only showed 28% recovery. After 24 h, the healing efficiency of diselenide increased to 76%, while disulfide PU only reached 43% [26]. NIPU featuring multiresponsiveness to humidity and temperature and healing properties by combining iminoboronate and boroxine chemistry was reported. NIPU samples showed complete scratch healing after 12 h at 70% relative humidity and at room temperature. The control NIPU containing nonreversible linkage displayed no healing when damaged and exposed to humidity for 12 h at room temperature [27].

In a previous manuscript, we reported the development of a bio-based 2K textile coating with excellent water barrier properties [28]. Nonetheless, despite the outstanding performance, the coating exhibited no barrier properties once damaged. Thus, a healable coating needs to be applied to extend the lifetime of the coated textile. However, although (self-)healing networks are extensively researched, none of the publications address the development of a bio-based healable PU for thin coatings such as textile coatings. Therefore, the composition of our previously reported 2K PU textile coating needed to be altered by replacing the high functional cardanol-based polyol with a self-healing diol without losing the water barrier properties. This report describes for the first time the synthesis and application of a new bio-based healable 2K PU on textiles based on dynamic covalent bonds with excellent barrier properties. Bio-based polyol, bio-based Schiff base with dual reversible linkage, hexamethylene diisocyanate (HMDI) were used as building blocks. HMDI was used as a building block, instead of Desmodur Eco N7300 in our previous 2K PU textile coating report, since Desmodur Eco N7300 resulted in highly crosslinked thermoset PU networks with lower chain mobility and no healing efficiency [28].

## 2. Materials and Methods

### 2.1. Materials

Bismuth neodecanoate, hexamethylene diisocyanate (HMDI), cystine and vanillin were purchased from Sigma-Aldrich. Transfer paper was sampled by Sappi. Tego Airex 900 (deaerator) and Dynasylan 1189 (adhesion promotor) were supplied by Evonik. Priplast 3172 was sampled by Croda. Woven polyester fabric (105 g/m^2^) was purchased form Concordia Textiles.

### 2.2. Synthesis of Schiff Base

The Schiff base was synthesized out of cystine and vanillin, which was based on a protocol published by Lee et al. with some minor modifications [21]. A solution of pH 10 was first prepared by adding 10 wt% of NaOH solution to 250 mL water. A total of 10 g of cystine and 12.66 g of vanillin were dissolved in this solution. After dissolving both cystine and vanillin, the pH was decreased, by adding 10 wt% NaOH until pH was 10. The mixture was stirred for 12 h at ambient temperature. During the reaction, the clear, colorless solution turned dark orange to brown, indicating Schiff base formation. The pH of the solution was adjusted to 7 using 1 M HCl to stop the reaction. The solution was then centrifuged for 8 min at 4000 rpm. The supernatant was then collected and dried overnight at 80 °C to remove water. The product was washed three times with 80 mL of chlorobenzene, followed by vacuum drying at 60 °C overnight. A brown solid residue was obtained with a yield of 55%.

### 2.3. Bio-Based Healable 2K PU Coating

The healable PU coatings were prepared by reacting Priplast 3172 (functionality: 2) and Schiff base (functionality: 4) with HMDI in a molar equivalent ratio 1:1. A total of 28 g of Priplast 3172 was mixed with 0.02 g bismuth neodecanoate. Subsequently, 6 g of Schiff base, dissolved in 5 g DMSO, was added followed by 0.3 g of Dynasylan 1189 and 0.3 g Tego Airex 900. Subsequently, 5.5 g hexamethylene diisocyanate was added to the polyol mixture. The 2K formulation was applied on transfer paper and a polyester fabric via knife over roll. The applied coating thickness was 50 µm. Two layers were coated, and each layer was cured for 2 min at 155 °C. A corresponding control 2K PU formulation without Schiff base was prepared (Table 1).

### 2.4. Characterisation

A Nicolet 6700 spectrometer from Thermofisher Scientific (Waltham, MA, USA) was used to record Fourier transform infrared spectra from 500 cm^−1^ to 4000 cm^−1^ to chemically characterize the PU networks.

An Elcometer 3086 Scratch Boy from Elcometer (Nieuwegein, The Netherlands) with a diamond scribe was used to make reproducible scratches in coatings. The force of the diamond scribe can be varied between 0 and 10 N. Healing of the coating was performed for 30 min at 90 °C (relative humidity in the closed space was 1%) and was visualized afterwards using a Field Emission Gun Scanning electron microscopy (FEG-SEM) (JSM 7600 F from Jeol Europe (Zaventem, Belgium)). Scratch healing was also observed via optical microscopy from Nikon (Dilbeek, Belgium).

Elongation at break and break at stress was determined using Instron electronic fabric tension tester according to ISO 13934-1 on 100 µm 2K PU films. The tension loading speed was 100 mm/min. Healing efficiency was calculated as the ratio between the elongation at the break of healed samples and the elongation at the break of undamaged samples.

Swelling experiments were performed in MEK to calculate the crosslinking density. The PU films were placed for 24 h in the solvent. After removal, residual solvent on the PU films was wiped off, before being weighed. From the weight of the swollen polymer (ws), the volume fraction of swollen polymer (Vp) can be calculated:

Vp = (wd/dp)/[(ws/ds) + (wd/dp)](1)

where wd is the dry weight of the polymer, and dp and ds are the densities of the polymer and solvent, respectively. The crosslink density (*n*) values were obtained from Vp with the Flory–Rehner equation:

−[ln(1 − Vp) + Vp + χVp2] = vs. *n* [ Vp1/3 − (Vp/2)] (2)

where vs. is the molar volume of the solvent and χ is the polymer–solvent interaction parameter, which can be found from the equation:

χ = (δ1 − δ2)2Vs/RT(3)

where R is a gas constant and T is the temperature (expressed in Kelvin), whereas δ1 and δ2 are the solubility parameters of the solvent and polymer.

The thermal behavior in air was analyzed via thermogravimetric analysis with a Q500 thermogravimetric analyzer from TA Instruments (Asse, Belgium) using standard parameters: a ramp rate of 10 °C/min from 30 °C to 600 °C.

Melting and glass transition temperatures of the PU coatings were determined via differential scanning calorimetry (DSC) analysis with TA Instruments Discovery DSC2500 from TA Instruments (Asse, Belgium). All samples were heated from −100 °C to 200 °C. Analyses were performed with a heating rate of 10 °C/min. The crystallinity (X_c_) of both control PU and healable PU was defined according to the equation:

X_c_ (%) = (ΔH_m_/ΔH_0m_) × 100% (4)

where ΔH_m_ is the melting enthalpy of PU and ΔH_0m_ is the melting enthalpy of 100% crystalline PU.

The dynamic mechanical properties of the samples (35 mm × 5.3 mm × 0.75 mm) were examined by a dynamic mechanical analyzer (DMA) using a TA Instruments 850 from TA Instruments (Asse, Belgium) in tensile mode from −100 °C to 120 °C at a heating rate of 3 °C/min at a frequency of 1 Hz. The creep recovery was also investigated by DMA using TA Instruments 850 at 90 °C and 0.1 MPa of stress.

The water barrier properties were measured with a Textest FX 800 apparatus from Artec Testnology (Kerkdriel, The Netherlands). A steadily rising water pressure (60 mbar/min) was applied on the coated surface, according to ISO 811 under standard conditions (20 °C and relative humidity of 65%), until penetration occurs in three places. The pressure at which penetration occurs at three places was set as the maximum hydrostatic pressure to which the textile can resist.

## 3. Results and Discussion

### 3.1. Structural Characterization of 2K PU Coatings

**Figure 1:**
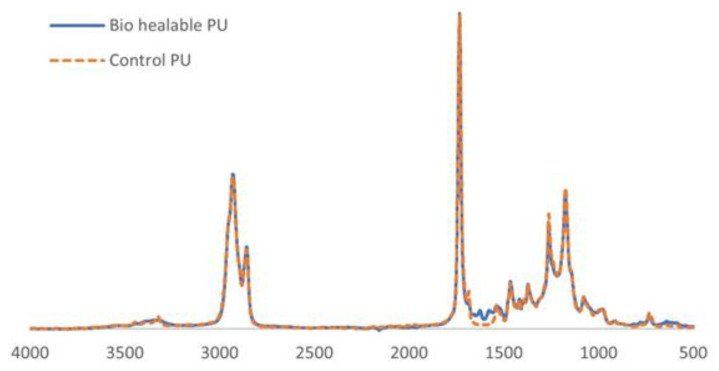
FT-IR spectra of bio-based healable and control PU.

The healable bio-based PU coating and the corresponding control PU were characterized by FT-IR (Figure 1). Both control and healable PU showed NH bands (3328 cm^−1^) and C=O stretching of the urethane and ester moieties, at, respectively, 1735 and 1683 cm^−1^. The signal at 1261 cm^−1^ is originating from C-O-C urethane vibrations, while C-O-C vibrations of the ester groups are found at 1174 cm^−1^. The peak at 1537 cm^−1^ is allocated to N-H bending and C-N stretching [29,30,31,32]. Contrary to the control PU, the healable bio-based PU exhibited 2 distinct IR bands at 1625 and at 1585 cm^−1^, which can be assigned to C=N stretch and aromatic C=C stretch of the Schiff base, respectively. Additionally, peaks at 3500–3400 cm^−1^ corresponding to the OH and COOH groups were not observed, indicating that the Schiff base was incorporated in the healable PU backbone. Swelling experiments were performed to study the crosslink density of both healable and control PU. However, the control PU dissolved in solvents acetone, MEK and THF. The crosslink density of the healable bio-based PU was calculated based on the swelling experiments in MEK and amounted to 505 m^3^/mol. The much higher solubility of the control PU compared to the healable bio-based PU in acetone, MEK and THF could indicate lower crosslinking density of the control PU.

### 3.2. Scratch Healing

**Figure 2:**
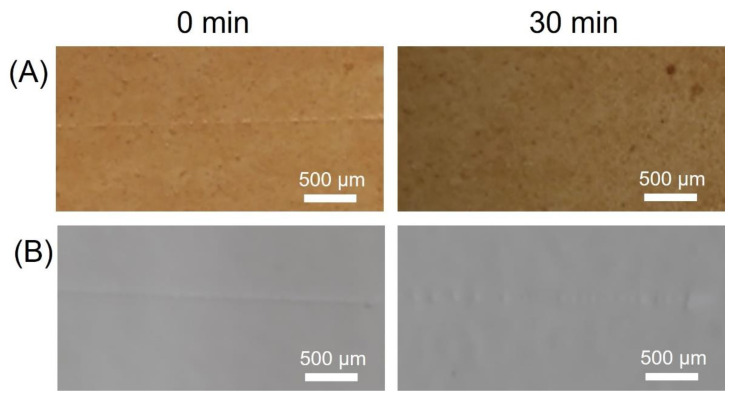
Images taken during scratch healing tests of healable PU (**A**) and control PU (**B**) coated textile after 0 and 30 min at 90 °C.

A scratch of 2 N was made on a healable and control PU coating. Optical images taken before and after healing are shown for both healable and control PU (Figure 2). Although the control PU healed slightly, due to the mobility of the PU chains at 90 °C, the scratch was still visible contrary to the healable PU coating, indicating that the healable PU coating exhibited superior healing.

**Figure 3:**
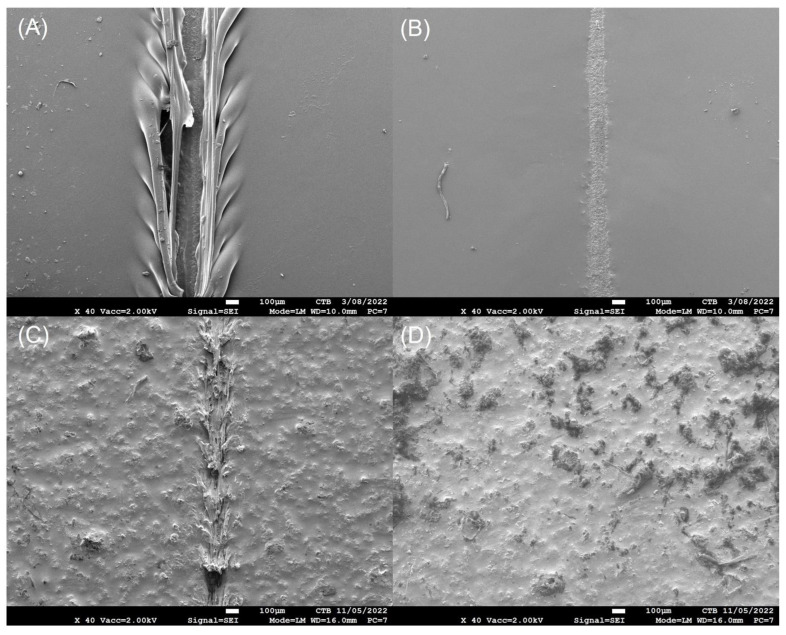
SEM analysis of scratched surfaces of control PU before (**A**) and after healing (**B**) and healable PU before (**C**) and after healing (**D**). Healing occurred for 30 min at 90 °C.

To confirm these results scratch healing was assessed by SEM analysis. Figure 3 shows SEM images of scratched surfaces of healable and control PU before and after healing. As indicated by microscopic images, both PU coatings showed narrowing of the scratch, but only in the case of the healable PU the scratch was closed and healed. Indeed, PUs possess dynamic hydrogen bonds between urethane moieties, resulting in the narrowing of the scratch, even in the absence of additional functional groups, but not complete crack closure [33]. In the case of the healable PU, the fusion of the scratched surface (scratch closure) could be ascribed to both hydrogen bonds as well as the dynamic exchange of imine and disulfide bonds after scratch closure. Further on, the SEM images show that the surface of the bio-based healable PU coating is much rougher compared to the smooth bio-based control PU coating. Due to the hydrophobic nature of both Priplast polyol and Desmodur Eco N7300, the Schiff base could not be mixed easily in the formulation. To improve the compatibility with the polyol and isocyanate, the Schiff base was dissolved first in DMSO prior to adding it to polyol and isocyanate. A pre-dissolution of the Schiff base in DMSO allowed the Schiff base to be mixed in the healable PU formulation. Our hypothesis is that phase separation (between Schiff base and polyol/isocyanate mixture) occurred due to partial solvent evaporation during curing of the 2K PU coating at 155 °C (which is close to the boiling temperature of DMSO), which contributed to surface roughness.

**Figure 4:**
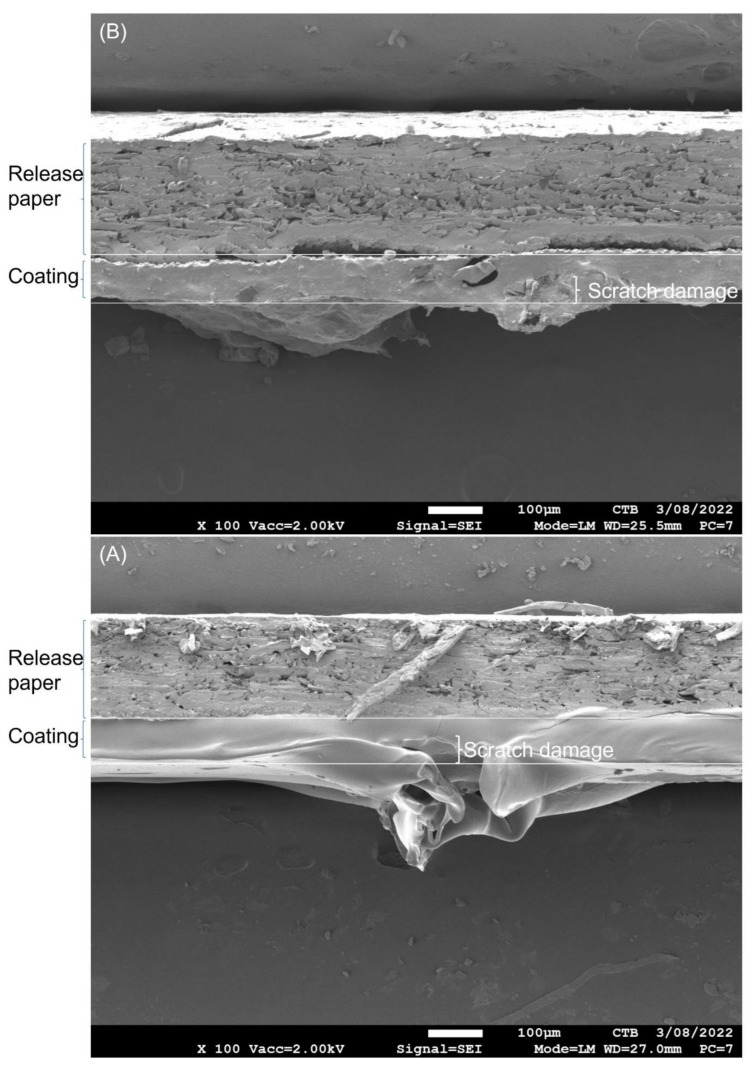
SEM cross-section analysis of scratched control PU (**A**) and healable PU (**B**) before healing.

SEM surface analysis seems to indicate that the scratch on the healable PU is shallower compared to the control PU. Therefore, a cross-section SEM analysis was performed to examine the depth of the scratch damage (Figure 4). The release paper with a coating is shown in the figure. Since the view is not perpendicular on the cross-section, the bottom surface of the release paper and the upper surface of the coating can be noticed and therefore the base and the top line of the coating is indicated in Figure 4.

For both control and healable PU, the depth of the scratch damage is the same. However, in the case of the healable PU, damaged PU coating parts seem to fill the crack (damaged parts bend into the crack) at room temperature, while this is not the case for the control PU (damaged parts bend outside the crack). This might be attributed to the lower melting point of healable PU compared to control PU, which results in a higher flow of the healable PU network resulting in PU parts that fill partly the crack. However, the consistency of the PU in the scratch of the healable PU is different compared to the non-damaged area since there is no healing or covalent linking yet at room temperature.

Assessment of the elongation was performed before and after the healing of different scratches for both the control and healable PU. The healable PU had a much higher initial tensile strength and elongation at break compared to the control PU, because of the incorporation of Schiff base as chain extender resulting in a higher molecular weight (Table 2). Table 3 exhibits the tensile strength after healing from scratches made with different forces. Healing efficiency was calculated as the ratio of the tensile strength of the healed samples and the maximum tensile strength of samples initially before scratching. Control PU exhibited low healing efficiency for scratches made with a 1 N force and exhibited no healing in the case of a scratch with a loading force of 2 N. The healing efficiency of healable PU was higher compared to the control PU, which is ascribed to not only the rearrangement of PU networks (scratch closure) but also the presence of reversible linkages based on disulfide and imine metathesis (scratch healing). Control PU showed only rearrangement of PU networks resulting in crack closure for small scratches but no intrinsic healing, resulting in lower healing efficiency. The healing efficiency after a 2 N scratch was also examined after 3 h of healing and amounted to 80%, which was slightly higher compared to the healing efficiency after a healing time of 30 min. The control PU also showed no healing after 3 h.

**Figure 5:**
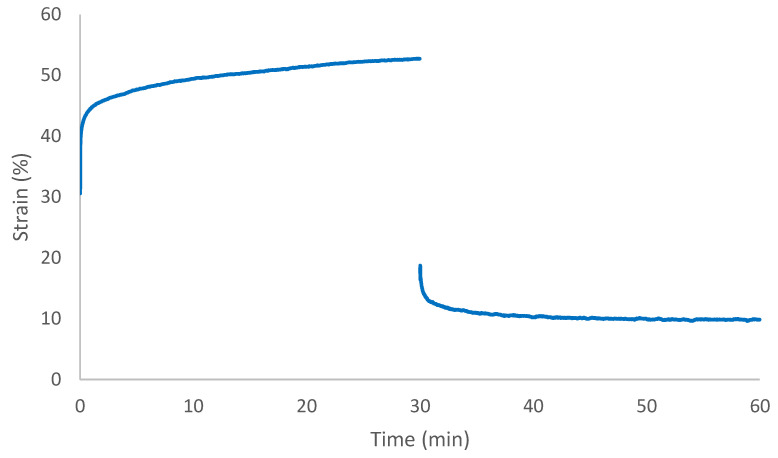
Creep and recovery test of healable PU at 90 °C.

Creep recovery experiments were performed at 90 °C (healing temperature) and applied a stress of 0.1 MPa for 30 min followed by 30 min of recovery. The results are presented in Figure 5. Creep recovery experiments could not be conducted on the control PU due to tearing of the sample because of the low mechanical properties. The healable PU had an incomplete strain recovery with a residual strain of 10%. The healable PU has a relatively high residual strain due to the faster rearrangement of networks via metathesis [21].

### 3.3. Thermal Behavior

**Figure 6:**
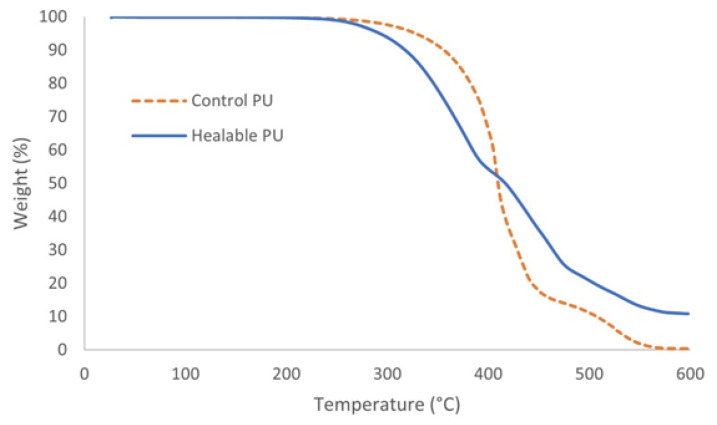
TGA curves of bio-based 2K PU coatings in air.

The thermal behavior of the developed coatings was examined via TGA (Table 4). The TGA curves are demonstrated in Figure 6. Both healable and control PU coatings showed similar thermal degradation; however, the healable PU exhibited a much higher weight residue at 600 °C compared to the control PU due to the higher sulfur content. Generally, degradation started above 200 °C. Three main degradation steps could be distinguished: decomposition of disulfide bonds and urethane groups, followed by dissociation of ester groups and linear aliphatic chains and finally breaking of C=C bonds [34,35,36].

**Figure 7:**
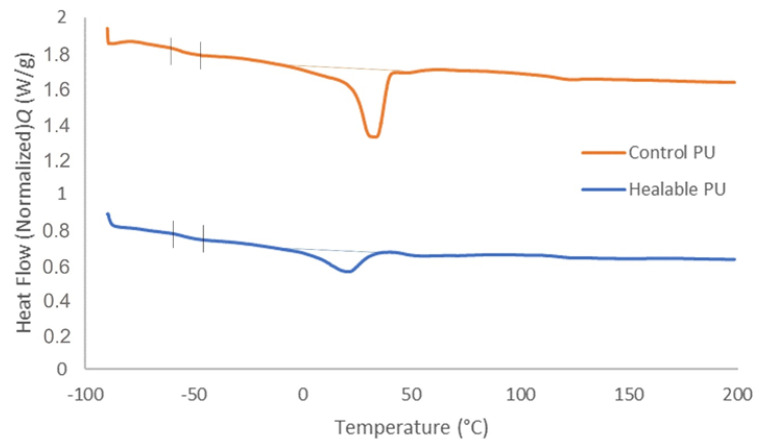
DSC curve of the bio-based 2K PU coatings.

Table 5 shows glass transition temperature (Tg) and melting temperature (T_m_), obtained via DSC. The healable PU showed slightly higher Tg than the control PU which could be attributed to the incorporation of dynamic covalent bonds (Figure 7). Compared to the control PU, the healable PU showed a lower melting point. Indeed, the healable PU is more branched due to the incorporation of the Schiff base, which had a functionality of 4 while the polyol Priplast 3172 has a functionality of 2, resulting in lower intermolecular forces in the healable PU and thus lower T_m_. The crystallinity degree of the healable PU is also significantly lower compared to the control PU since the healable PU is more branched. Indeed, branches in a polymer decrease the ability of close packing of the chains, thereby reducing the crystallinity of the polymer [37,38].

**Figure 8:**
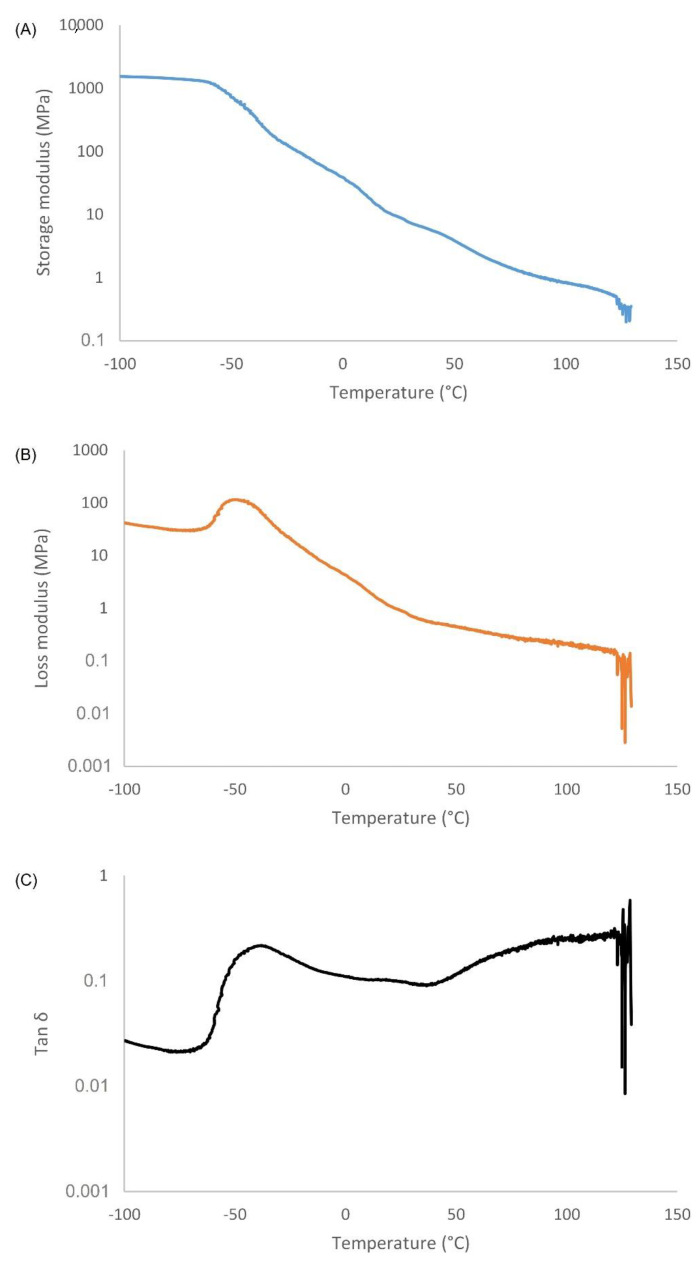
Dynamic mechanical properties of PU networks in DMA measurements: (**A**) storage modulus (E′) versus temperature; (**B**) loss modulus (E″) versus temperature and (**C**) tan δ versus temperature.

The results of the DMA analysis are presented in Figure 8. The Tg is defined as the onset of the E′ drop and was found to be −50.3 °C, which corresponds well with the Tg value found with DSC. The storage moduli (E′) of the healable PU was steady below Tg and the tan δ peak exhibited a lower value than above Tg due to restricted chain mobility. The healable PU showed a significant drop of E′ above Tg due to the rearrangement of PU chains and breaking and reforming of the dynamic covalent bonds [39].

### 3.4. Performance of the Bio-Based 2K PU Coating

The 2K PU formulations were applied on woven fabric and the water barrier initially and after healing of 2 N scratch was measured (Table 6). The fabrics coated with the healable PU showed excellent water barrier characteristics initially, while polyester coated with the control PU had lower water barrier properties. After making a 2 N scratch in the coating, the resistance to hydrostatic pressure dropped to significantly lower values for both coated fabrics. However, after healing (30 min at 90 °C) the resistance to the hydrostatic pressure of the healable PU-coated fabric increased again to the maximum value of 1000 mbar, indicating that healing of the coating also restored the barrier properties. Contrary to the healable PU-coated fabric, the control PU-coated fabric showed no significant increase in the water barrier properties after healing. This indicates that the resistance to the hydrostatic pressure of the control PU-coated fabric is not restored after healing and that the control PU has no healing effect.

## 4. Conclusions

Often microcapsules are added as a healing additive in a textile coating. Upon a scratch in the coating, the microcapsules will be ruptured, and the healing agents will be released to close the crack. However, this is a non-reversible process. Therefore, reversible healing is being looked at. The effectiveness of supramolecular polymers for commercial PU textile coatings was already examined. However, the supramolecular polymers needed a higher temperature to induce healing at a short time and did not restore the water barrier properties [40]. Therefore, a bio-based Schiff base, having reversible disulfide and imine bonds, was synthesized and incorporated in a bio-based 2K PU formulation to impart healing properties. The formulation was coated on a polyester textile. The FT-IR analysis indicated that the healable PU coating incorporated the Schiff base and contained no remaining isocyanate. The residual strain of the healable PU amounted to 10%, due to bonds exchange leading to quicker rearrangement of networks. The healable bio-based PU coatings had a healing efficiency of 75% based on the recovery of tensile strength after healing, while control PU coatings did not heal. Furthermore, the water barrier properties of the healable bio-based PU coating were completely restored after healing contrary to control PU coatings. The incorporation of Schiff base is therefore concluded to provide excellent healing properties due to the presence of reversible imine and disulfide bonds. Because of the very high water barrier, the developed healable bio-based 2K PU is suitable as a coating for waterproof textiles. The healable PU with Schiff base allows recovering of the water barrier properties of thin textile coatings at a relatively low temperature and in a short time.

## Figures and Tables

**Table 1:** Composition of bio-based healable and control 2K PU coatings (amounts are expressed in grams).

	Priplast 3172	Schiff Base	DMSO	Bismuth Catalyst	HMDI	Dynasylan 1189	Tego Airex 900
Healable PU	28	6	5	0.02	5.5	0.3	0.3
Control PU	28	-	5	0.02	1.69	0.3	0.3

**Table 2:** Initial tensile strength and elongation of healable and control PU.

	Tensile Strength (MPa)	Elongation at Break (%)
Healable PU	1.11 ± 0.58	384.3 ± 34.0
Control PU	0.46 ± 0.10	12.0 ± 1.3

**Table 3:** Tensile strength of healable and control PU after healing. *: Tensile strength could not be measured since the scratch was not healed.

	Tensile Strength (MPa)		Healing Efficiency (%)	
	Healed 1 N Scratch	Healed 2 N Scratch	Healed 1 N Scratch	Healed 2 N Scratch
Healable PU	0.89 ± 0.01	0.83 ± 0.08	81%	75%
Control PU	0.20 ± 0.05	- *	44%	-

**Table 4:** Overview of T_5%_ and weight residue at 600 °C for the bio-based 2K PU coatings.

	T_5%_ (°C)	Weight Residue (%)
Healable PU	292.35	10.835
Control PU	327.81	0.389

**Table 5:** Tg, T_m_ and X_c_ of the healable and control bio-based 2K PU.

	Tg (°C)	T_m_ (°C)	X_c_ (%)
Healable PU	−54.6	20.5	13.8
Control PU	−57.5	32.9	28.2

**Table 6:** Water barrier properties of bio-based 2K PU coated polyester fabric initially, after scratch damage and after healing.

	Initial	2 N Scratch	After Healing
Healable PU	≥1000	532	≥1000
Control PU	702	252	276

## Data Availability

The data presented in this study are available on request from the corresponding author.
